# Neurovascular coupling: *in vivo* optical techniques for functional brain imaging

**DOI:** 10.1186/1475-925X-12-38

**Published:** 2013-04-30

**Authors:** Lun-De Liao, Vassiliy Tsytsarev, Ignacio Delgado-Martínez, Meng-Lin Li, Reha Erzurumlu, Ashwati Vipin, Josue Orellana, Yan-Ren Lin, Hsin-Yi Lai, You-Yin Chen, Nitish V Thakor

**Affiliations:** 1 Singapore Institute for Neurotechnology (SINAPSE), National University of Singapore, 28 Medical Drive, #05-COR, Singapore 117456, Singapore; 2 Department of Anatomy and Neurobiology, University of Maryland School of Medicine, 20 Penn street, HSF-2, Baltimore, MD 21201, USA; 3 Department of Electrical Engineering, National Tsing Hua University, No. 101, Sec. 2, Kuang-Fu Rd, Hsinchu 300, R.O.C, Taiwan; 4 Department of Emergency Medicine, Changhua Christian Hospital, 135 Nanshsiao Street, Changhua 500, R.O.C, Taiwan; 5 Department of Physical Medicine and Rehabilitation, Chang Gung Memorial Hospital and Chang Gung University, Taoyuan 333, R.O.C, Taiwan; 6 Department of Biomedical Engineering, National Yang Ming University, No.155, Sec.2, Linong St, Taipei 112, R.O.C, Taiwan; 7 Department of Biomedical Engineering, Johns Hopkins University, Traylor 701/720 Rutland Ave, Baltimore, MD 21205, USA

**Keywords:** Neurovascular coupling, Cerebral neuroimaging, 2-photon microscopy, Laser speckle contrast imaging, Voltage sensitive dye imaging, Functional photoacoustic microscopy, Functional near-infrared spectroscopy

## Abstract

Optical imaging techniques reflect different biochemical processes in the brain, which is closely related with neural activity. Scientists and clinicians employ a variety of optical imaging technologies to visualize and study the relationship between neurons, glial cells and blood vessels. In this paper, we present an overview of the current optical approaches used for the *in vivo* imaging of neurovascular coupling events in small animal models. These techniques include 2-photon microscopy, laser speckle contrast imaging (LSCI), voltage-sensitive dye imaging (VSDi), functional photoacoustic microscopy (fPAM), functional near-infrared spectroscopy imaging (fNIRS) and multimodal imaging techniques. The basic principles of each technique are described in detail, followed by examples of current applications from cutting-edge studies of cerebral neurovascular coupling functions and metabolic. Moreover, we provide a glimpse of the possible ways in which these techniques might be translated to human studies for clinical investigations of pathophysiology and disease. *In vivo* optical imaging techniques continue to expand and evolve, allowing us to discover fundamental basis of neurovascular coupling roles in cerebral physiology and pathophysiology.

## Introduction

Cerebral blood flow (CBF) is vital to the normal brain. The average CBF in humans is approximately 55 ml per 100 g of brain tissue per minute [1]. Between 700 and 800 ml of the normal cardiac output of 5 l/min is used to maintain membrane potentials and reverse ion fluxes from action potentials in the brain [2]. More blood flow is needed when active neural processes increase energy requirements. Blood flow correlates precisely with the regional energy utilization in the brain matter [3]. That is, the cerebral circulation must maintain a constant and adequate blood flow for the brain to function [1]. The cerebrovascular system has several mechanisms to ensure the correct blood circulation to meet specific energy demands and prevent harmful fluctuations due to changes in arterial pressure. This autoregulation of the cerebrovascular system involves several different blood vessels with contractile elements, such as layers of smooth muscle cells, which may control the diameter of the vessel and the CBF. In contrast to other organs, the parenchymal flow in the brain is controlled entirely outside of the organ. Two-thirds of the vascular resistance in the brain is due to large cerebral arteries and pial vessels [4]; non-pial vessels are responsible for the remaining one-third of vascular resistance [4].

Furthermore, the brain capillary system is highly heterogeneous. The brain uses an additional mechanism, hyperaemia, to increase the flow of blood to the regions in which neurons are active. According to this mechanism, neurons and astrocytes directly regulate the local blood flow within the capillaries, resulting in local neurovascular coupling. Initially, it was proposed that the local increase in blood flow upon neuronal activation was caused by a metabolic signal, such as a decrease in either the O_2_ or glucose concentration or accumulation of CO_2_. However, recent experiments have shown that a neurotransmitter-mediated mechanism that involves astrocytes is responsible for local flow regulation [5]. According to this, upon glutamate activation, nitric oxide (NO) and arachidonic acid derivatives are released from neurons and astrocytes, respectively, upon glutamate activation. Although this hypothesis was initially debated, there are extensive studies showing that the final effect of these substances depends on the local O_2_ concentration and differs between brain regions [6,7]. Furthermore, this mechanism of local vascular flow regulation may affect not only smooth-muscle-dependent arteriole contraction but also the intrinsic contraction of the pericytes, which are supporting cells of capillaries, playing a key role in both neurovascular coupling and the maintenance of the blood–brain-barrier (BBB) [6,7].

Neurovascular coupling and the correlation between neural activity and the CBF can be exploited to examine both normal brain function and the pathophysiology of the cerebrovascular system. Optical imaging techniques provide the necessary tools to accurately detect and analyze regional hyperemic changes. This review will present several important optical imaging techniques that are used for *in vivo* measurement of functional hyperemia, including 2-photon laser scanning microscopy (TPLSM), laser speckle contrast imaging (LCSI), voltage-sensitive dye imaging (VSDi), functional near-infrared spectroscopy (fNIRS) and functional photoacoustic microscopy (fPAM). Due to space constrictions, we refer to other reviews addressing functional magnetic resonance imaging (fMRI) [8], intrinsic signal optical imaging (IOS) [9,10] and other techniques [11,12]. In the conclusion of this review, we discuss the possible applications of these *in vivo* techniques in basic and clinical human studies, especially in techniques that can be administered intraoperatively.

## Examination of cerebral neurovascular and neurometabolic functions using optical imaging techniques

The question of how to examine the relationship between neural, vascular, hemodynamic and metabolic responses using optical imaging techniques has not been answered comprehensively. The relationship between optical signals of cerebral blood oxygenation (SO_2_) and neuronal activity is complex. The response depends on the coupling of neurovascular functions to the corresponding cerebral neurometabolic functions of tissues and cells. Optical techniques to probe functional brain activity are known to produce reliable results. Furthermore, the specific characteristics of these optical signals are sensitive to the corresponding cerebral vascular physiology and structure.

Different kinds of optical signals reflect different neuronal activity in different parts of the brain: changes in blood oxygenation, local blood circulation, tissue concentration of oxy- and deoxyhemoglobin. Neural and glial [13-15] cell volume changes are frequently associated with alterations in different components of the optical signals. Since brain metabolism, local blood circulation and cell volume (swelling) reflect neural activity, we can reasonably assume that the different kinds of brain optical imaging can be applied to help understand the localization of neural activity.

**Figure 1:**
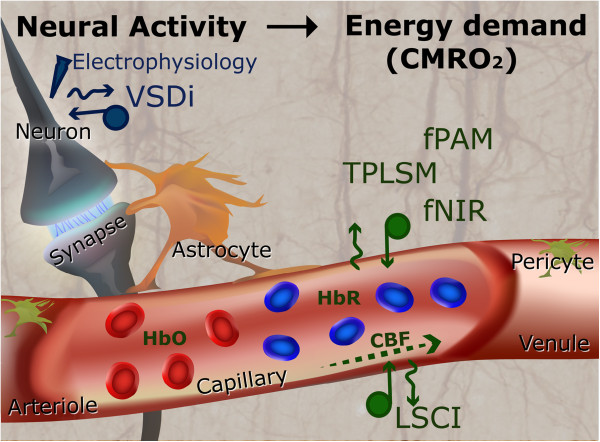
**An overview of the complete neurovascular model which assumes that the optical signals directly correspond to changes in the CMRO**_**2**_**.** Normally neural activity is accompanying by changes in the local oxygenation. The relationship between neural activity, oxygen metabolism, and hemodynamic can be studied by variable imaging techniques (including VSDi, LSCI, fPAM, TPLSM and fNIR) in combination with local field potential recordings.

Figure 1 provides a schematic representation of the neurovascular coupling model [16,17] most commonly used to characterize the vasomotion and metabolic responses in the brain. This model assumes that the optical signals directly correspond to changes in the cerebral metabolic rate of oxygen (CMRO_2_). Several researchers have proposed a set of equations to describe the response of the arteries and capillaries in neurovascular coupling [18-20]. Neurons can be triggered by unique stimulation events to produce corresponding evoked potential signals (EPSs). These EPSs are directly correlated with the responses of the vascular and oxygen transport models [21].

The response of the vascular model also affects the CBF response, which drives changes in cerebral blood volume (CBV) and total of hemoglobin (HbT) (includes oxyhemoglobin (HbO) and deoxyhemoglobin (HbR)). Existing optical imaging techniques for probing hemodynamic changes are based on either the absorption model (*i.e.*, TPLSM, fNIRS and fPAM), the speckle model (*i.e.*, LCSI) or activity-dependent fluorescence model (VSDi) (Table 1). The optical absorption model can be applied to experimental measurements to predict HbR and HbO directly *via* the oxygen transport model because of the unique contrast witnessed in optical signals. In addition, the oxygen level in the blood vessels can be modeled using the principles of mass balance based on the CBF changes predicted from the vascular model for oxygen consumption metabolic coupling. On the other hand, the speckle model is directly related to changes in CBF because speckles are the result of interaction of coherent light with the scatters (mainly red blood cells (RBCs)) in the blood vessels. In this section, we will describe how each of these imaging techniques works, as shown in Table 1, and as well as elaborate upon their applications.

**Table 1:** Comparison of spatial and temporal resolution, penetration depth and limitations of different optical imaging techniques

	**Depth**	**Temporal and spatial resolutions**	**Optical source**	**Contrast agent**	**Limitations**
**2-Photons microscopy**[12,22-25]	Up to 1 mm	Spatial resolution is up to 1 μm. The temporal resolution is variable and determined by beam-scanning methods. In specially developed high-speed 2-photon imaging systems temporal resolution can reach a few μs.	Two-photon excitation wavelengths are typically around twice the usual fluorescent excitation wave- lengths. Most fluorescent probes have excitation in the 350–650 nm range, whereas the excitation laser is in the ~700–1300 nm range.	Delivered from outside or genetically encoded fluorescent probes. Since a fluorescence probe can be treated with voltage- or calcium- sensitive dye, fluorescence antibodies or any kind of fluorescence biomarkers	The temporal resolution of the technique is defined by the property of the imaging setup. High power laser causes photo-bleaching and even destroys cells. The method is invasive and applicable only for relatively small regions.
**Laser speckle contrast imaging**[26-34]	0.5 - 1.0 mm	Up to 10 μm, no axial/depth resolution. The temporal resolution is limited by laser scanning methods and imaging of small areas can reach few tens μs. The temporal resolution is determined by the setup and can reach up to 1 μs.	Laser wavelength usually ranging from 400 to 1200 nm but very variable and is determined by the experimental goal	No requirement for a chemical agent	Invasive. The temporal resolution is mainly defined by the parameters of the CCD camera
**Photoacoustic microscopy**[35-42]	Up to a few centimeters, but high resolution can be reached only up to a few mm in depth.	Up to 1 μm, but depends on imaging depth, acoustic transducer and optical focusing. Temporal resolution is from milliseconds to sub-milliseconds.	Laser wavelength, depending on the target. For example: 570 nm is sensitive to HbT.	Usually doesn’t need any contrast agents but can be combined with different biomarkers.	The temporal resolution is restricted by the technical characteristics of the laser scanning system. Without the use of contrast agents it is applicable for the monitoring of the cerebral blood flow and oxygenation, but not for neural and metabolic activity.
**Near-infrared spectroscopy**[9,10,43-46]	Up to few centimeters transcranially; 1–2 mm in an open brain	From 2–3 cm in case of human transcranial research to ~0.1 mm in open brain animal experiments. The temporal resolution can be as high as 1 ms.	Monochromatic near-infrared light source, usually 700–1700 nm.	Based on the difference in the light absorption of HbO and HbR and doesn’t need any contrast agents.	Applicable trans-cranially as well as in open brain imaging, human research, clinical practice and animal experiments.
**Voltage-sensitive dye imaging**[10,23,24,47,48]	Up to ~ 1 mm	The spatial resolution is determined by the optical system and usually limited to 50 microns. However, in combination with 2-photon imaging it can reach 1–2 microns. The temporal resolution is limited by VSD properties and imaging rate and can reach milliseconds or even submillisecond resolution.	Monochromatic light in case of conventional imaging and long wavelength laser in 2-photon imaging	Voltage-sensitive dye – the chemical compounds which change their optic properties in response to the changes in the neural membrane potential.	Invasive, application is limited due to toxicity and photo-bleaching. The temporal resolution is defined by the CCD camera and can reach 1 ms or less.

### 2-photon laser scanning microscopy

**Figure 2:**
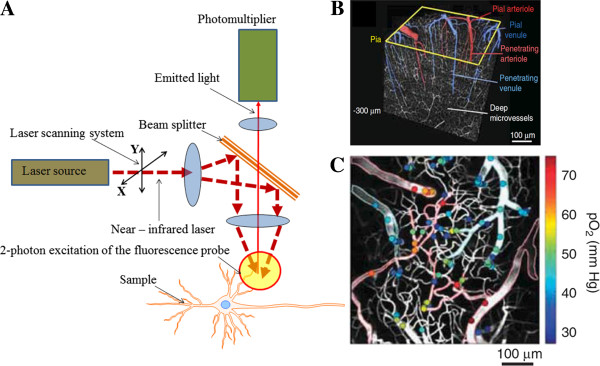
**(A) Main parts of the TPLSM **[22]**.** 2-photon excitation can have advantages for 3D imaging of the relatively thick, up to 1 mm and more objects in vitro as well as in vivo. (**B**) Three-dimensional TPLSM high-resolution image [25]. (**C**) Intravascular oxygen could be measured over various depths of cortex by TPLSM. The color bar shows the calculated partial pressure of oxygen at the measured location [25].

Several advances in microscopy techniques have allowed examination of capillary flow based on the gaps between RBCs during their movement along the blood vessel. These techniques initially involved the use of a CCD detector [49], then fluorescence microscopy [9] and, finally, confocal laser scanning microscopy [50,51]. However, the strong light-scattering associated with brain tissue that occurs when using these techniques prevented cellular and subcellular resolution in the intact brain. High-resolution imaging of brain cell morphology and function *in vivo* was not possible until TPLSM (Figure 2A) was developed by Denk et al. [22]. The description of the physical properties of the two-photon absorption phenomenon is beyond the scope of this review. We thus refer to previous literature for a detailed description [52,53].

TPLSM has been employed to measure vascular changes in single vessels *in vitro*[54-59] and *in vivo*[60-64]. Neuronal activity can be imaged simultaneously using calcium indicators [65]. The ability to image both neuronal and glial calcium transients is important for neurovascular researches because both neurons and glia have been implicated in vasculature communication [54,56,57,63,66]. Furthermore, functional changes in calcium levels can also be detected in arteriolar smooth muscle, and these changes appear to coincide with active changes in vascular diameter [67]. Therefore, TPLSM can be used to observe neurons, glia and the vessels which control blood flow through a combination of calcium imaging and direct blood flow measurements (Figure 2B). This approach is expected to aid in the identification of cellular elements which control vascular responses under normal conditions and, possibly, the mechanisms which lead to failure under pathological conditions.

As well known, the essence is that a fluorophore – Ca^2^+ − or voltage-sensitive dye, fluorescent proteins, or other sort of fluorescence markers is excited by the absorption of two photons at the same time, each of which brings only part of the energy required for fluorophore excitation. Different fluorophores may have different emission wavelength that is allow to generate separated 2D and 3D images for the different sorts of cells.

Two of the main advantages of TPLSM over single-photon microscopy techniques are its higher resolution and ability to image greater depths (up to 600 μm) below the cortical surface [62,68]. In the 2-photon absorption process, the fluorescence signal depends nonlinearly on the amount of excitation by light. Therefore, fluorescence generation is localized to the focal spot. In contrast to confocal microscopy, resolution is achieved by limiting the excitation volume at the focal point, instead of limiting the detection of a photon with a pinhole. This technique minimizes tissue damage caused by photochemical interactions. Furthermore, the excitation light required to combine two photons to excite a fluorophore has a longer wavelength (700–1,000 nm) compared with the light used in single-photon microscopes. Therefore, the resulting scattering is significantly reduced, and greater depths can be reached. However, because the resolution scales inversely with the wavelength, the resolution decreases progressively as depth increases [69].

Recent advances in TPLSM have allowed simultaneous measurement of the activity of single, visually identified blood vessels and visualization of neuronal and glial activity using calcium indicators [11,12,23]. TPLSM can be employed to calculate vascular dynamics at the single-vessel level by measuring the diameter of the vascular lumen and RBC speed (Figure 2C). A common technique to visualize blood vessels utilizes space-occupying high-contrast molecules, such as fluorescent dye-dextran conjugates (e.g., fluorescein isothiocyanate, FITC) [70]. Continuous line scans perpendicular to the vessel axis can be conducted to measure changes in vessel diameter over time, and RBC speed can be calculated from repeated line scans along the axis of the vessel lumen. The movement of RBCs over the background fluorescence of dextran-containing fluid generates streaks in the space-time stack image. The blood flow can then be calculated as a product of the vessel cross-section area and RBC speed [71].

### Laser speckle contrast imaging (LSCI): a powerful tool for probing cerebral blood flow

**Figure 3:**
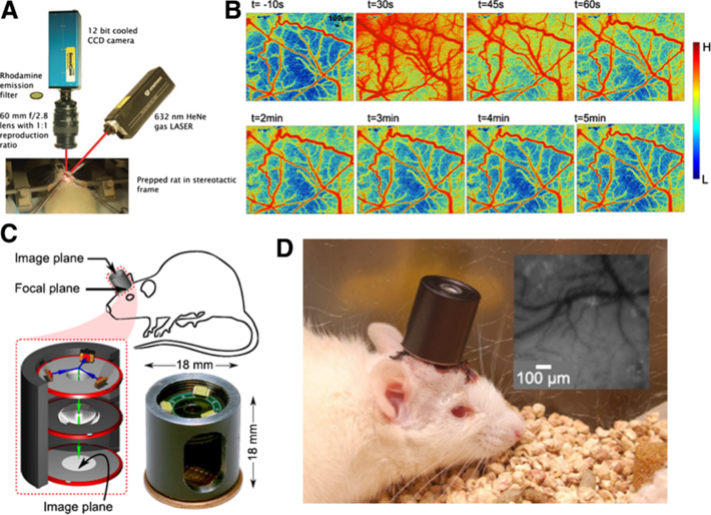
**(A) Setup of the experimental LSCI **[72]**.** (**B**) Images displaying the vascular responses during and after electrical stimulation of peripheral trigeminal nerve fibers [28]. (**C**) A schematic drawing of the integrated imaging microscope shown that the incident and reflected light paths in blue and green, respectively; and a photograph of the assembled device [73]. (**D**) This integrated imaging microscope can be used for untethered cortical imaging in freely-moving animals [73].

LSCI is a very practical and economic imaging tool used to visualize fine microvasculature and perfusion dynamics (Figure 3A). Physically, LSCI is a powerful imaging method based on light absorption and scattering and focuses on interpreting the speckle pattern phenomenon - intensity patterns produced by the interference of a set of light waves.

LSCI is a non-scanning imaging modality that is capable of monitoring a wide field of view with a high temporal resolution. LSCI utilizes RBCs to induce speckle contrast of laser light, which is inversely related to CBF [26]. The details of the theory and methodology [27] underlying the LSCI technique can be found in previous reports [26,28,29]. However, LSCI can only be used to generate information from single vessels that are located on or close to the cortical surface due to its limited penetration depth and poor depth resolution [26,28,29]. Nevertheless, LSCI is a popular imaging tool because of its ability to directly assess cerebral flow velocities with an excellent resolution, without the need for an exogenous contrast agent.

The LSCI technique has been applied by many researchers to study the spatiotemporal evolution of cortical hemodynamic patterns in response to functional stimulation. Li *et al*. developed an LSCI system with a high resolution (6.7 μm × 6.7 μm) to characterize the CBF and corresponding vasomotor response that occurred in response to electrical stimulation of the rat peripheral trigeminal nerve [28] (Figure 3B). The trigeminal neural system is an important factor in the pathogenesis of migraines [74], and understanding the relationship between this system and migraine *via* the LSCI technique will be useful for further examining disorders of the neurovascular system, especially for exploring their underlying cellular and molecular basis. Moreover, Bouchard *et al.* proposed an LSCI system with a lower overall cost compared with the LSCI system currently employed in laboratory settings. This low-cost LSCI system enables simultaneous visualization of HbT, HbO_2_ and Hb dynamics within single vessels in response to forepaw stimulation [75]. LSCI has proven to be an important tool for neuroscience research, presenting excellent spatial and temporal resolutions and capabilities that extend beyond the visualization of cerebral functional and structural hemodynamic patterns.

There is great interest in studying neurovascular responses of animals in an awake state because recent studies have shown that anesthesia may alter the responses of neural circuits. Most of the LSCI systems that have been developed to monitor CBF can be used only on anesthetized animals. Therefore, a novel LSCI device that can examine CBF changes in small anesthetized animals is needed. However, in designing such a device for use in freely moving animals, there are several major challenges: 1) the imaging device must be lightweight, and the equipment response must be rapid; 2) anti-motion artifacts need to be considered in freely moving animals; and 3) the wireless charge and signal transmission must be robust, especially for behavioral studies. How to overcome the above-mentioned issues using technology to minimize LSCI systems into a reliable device for use in freely moving animals is an issue of great interest and importance. Recently, Miao *et al*. proposed a miniature LSCI imager that weighs approximately 20 g and can image CBF changes in freely moving animals [30]. This miniature LSCI imager includes an image sensor, a light source, an optical lens and a data acquisition board and can generate real-time CBF images with a high spatiotemporal resolution in freely moving animals.

Murari *et al*. proposed an integrated miniature LSCI microscope that allows several optical techniques to be applied (*i.e.*, reflectance, spectroscopy, speckle and fluorescence imaging) [73] (Figure 3C- 3D). This novel lightweight LSCI microscope can image cortical CBF changes in mobile rats. In the future, widespread use of the LSCI technique to image awake and active animals will provide critical information about currently unknown cerebral neuroscience issues in these animals (Figure 3D). Moreover, this technique will offer the advantage of allowing physiological processes and structures to be studied in freely moving animals.

### *In vivo* VSDi: direct imaging of neural activity and neurovascular coupling

**Figure 4:**
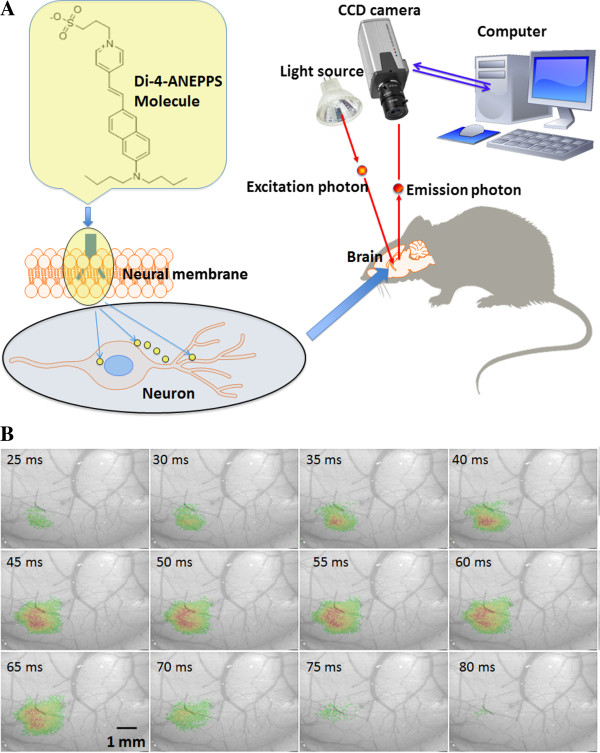
**(A) Main principle of the VSDi system.** Commonly used voltage sensitive dye di-4-ANEPPS, or other dyes, are able to provide measurements of membrane potential of single neurons or large neuronal populations. Dye molecules are localized in membranes with their hydrophobic tails and transduce membrane potentials changes into the optic features. (**B**) A VSD optical images showing mouse whisker stimulation fluorescence changes [78]. The stimulus onset was at the beginning of first frame number one, the time after stimulus onset is indicated at the bottom left corner of each image.

Over the past several decades, various imaging approaches have been developed to investigate both individual and network neuronal properties in living organisms. Imaging techniques such as fMRI and IOS are used to detect changes in brain tissue oxygenation levels in response to neural activity. VSDi (Figure 4A) can achieve a much higher spatial and temporal resolution compared with these techniques. This approach is particularly useful for generating functional images of neural circuit dynamics in superficially located brain structures such as the neocortex [76]. VSDi is based on the electrochemical properties of the neural membrane. Voltage-sensitive dye molecules change the level of light absorbance or fluorescence in proportion to the membrane potential. Therefore, they allow visualization of neural activity with a high temporal (less than 1 millisecond) resolution [77].

In VSDi, the optical signal is determined by two components: 1) the amplitude of the changes in the membrane potential and 2) the size of the membrane surface stained with the voltage-sensitive dye. In the neocortex, the dendrites cover a larger area than the neuronal soma; therefore, the fluorescence signal mostly reflects postsynaptic potentials, rather than action potentials [76]. The response time of the voltage-sensitive dye molecules is quite short but is longer than the time over which a membrane potential changes. For some probes, the response time is in the microseconds and shows a linear correlation with the membrane voltage. The two main classes of voltage-sensitive dyes can be differentiated based on their chemistry: the first class of dyes undergoes changes in absorbance and the second class changes in fluorescence [79].

The excitation and emission wavelengths of the two main classes of voltage-sensitive dyes are also different. For *in vivo* research, these wavelengths should show minimal overlap with hemoglobin absorption; however, this consideration is not critical for *in vitro* imaging. During *in vitro* imaging, the sample is illuminated by a monochromatic excitation light, and the fluorescent signal is recorded by a CCD camera or a photodiode array through appropriate optic filters. The temporal resolution of VSDi is limited by the technical properties of the imaging setup, whereas the spatial resolution is determined by the optical scattering of the brain tissue and usually cannot exceed a few tens of microns [79]. Spatial resolution can be improved by applying VSDi in combination with either confocal laser scanning microscopy for *in vitro* experiments or multiphoton microscopy for both *in vivo* and *in vitro* experiments [24].

Due to chemical toxicity, VSDi is not suitable for use in clinical practice. However, VSDi is a powerful technique for animal experiments, including visual [47], auditory [80] and somatosensory [48,81] research (Figure 4B). This field of brain imaging will undoubtedly benefit from the development of genetically encoded fluorescent voltage-sensitive probes, such as voltage-sensitive dye proteins (VSDP) [82]. These genetically engineered proteins allow the analysis of rapid electrical signals in neural populations *in vitro* as well as *in vivo*. Encoded fluorescent probes can also be targeted to selected neurons in different parts of the brain.

### Neurovascular photoacoustic microscopy

**Figure 5:**
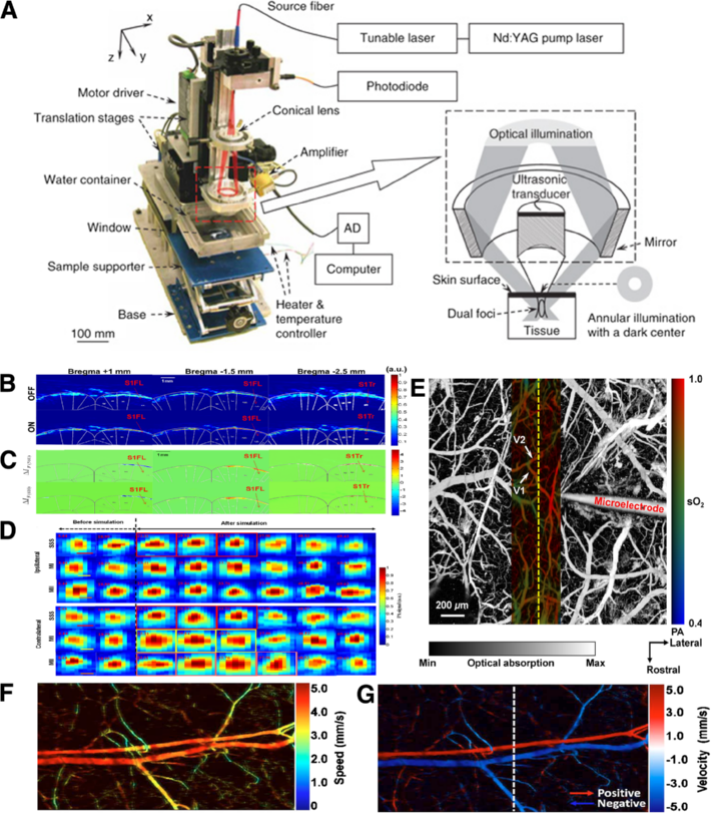
**(A) AR-PAM system **[85]**.** PA imaging to probe changes in cortical blood volume (**B**) and oxygenation (**C**) without a labeling agent [40]. (**D**) *In vivo* transcranial imaging of changes in HbT in single cerebral cortex vessels in small animals [37]. (**E**) Cerebral hemodynamic responses to neuronal activities induced by direct cortical electrical stimulation in rats; V1, V2 – observed blood vessels [86]. (**F**-**G**) A method to measure transverse blood flow was employed PA Doppler, showing the (**F**) speed and (**G**) velocity with directions [87].

Photoacoustic (PA) imaging is an optical absorption-based hybrid imaging technique that combines the advantages of optical and ultrasound techniques to provide a high optical absorption contrast and ultrasonic spatial resolution at a penetration depth of up to several centimeters (Figure 5A). This technique visualizes the high optical absorption contrast of biological tissues, instead of the low acoustic scattering contrast. The spatial resolution of PA imaging is determined primarily by the characteristics of the ultrasound transducer, such as center frequency, bandwidth, and numerical aperture, rather than by optical diffusion (as in optical imaging) [83]. Ultrasonic detection improves the spatial resolution of PA imaging up to few microns [83] and can also improve it up to even one micron [84]. Moreover, PA imaging is uniquely sensitive to blood *in vivo* due to the high intrinsic optical absorption of blood relative to other biological tissues [40]. PA imaging of *in vitro* and *in vivo* blood samples has shown that this technique can assess relative changes in the concentrations of both oxyhemoglobin and deoxyhemoglobin [40]. Using appropriate and distinct PA excitation wavelengths, it has been demonstrated that changes in HbT, CBV and SO_2_ can be probed independently. For example, photoacoustic signals generated at 560 or 600 nm are sensitive to SO_2_ changes, whereas the signals at 570 nm, an isosbestic point of the molar extinction spectra of oxy- and deoxy-hemoglobin, are instead insensitive to SO_2_ but on the other hand can be used to image changes in HbT and CBV [40].

A reflection-mode PA microscopy (PAM) technique involving dark-field illumination has been proposed by Maslov, *et al.*[84] (Figure 5A). PAM can be further divided into optical- (OR-PAM) and acoustic-resolution PAM (AR-PAM), depending on the resolution and penetration preference for an optical or ultrasonic focus in experiments, respectively. OR-PAM provides an imaging resolution at the cellular level (i.e., ranging from a few hundred nanometers to a few micrometers). At depths beyond the optical diffusion limit and up to a few millimeters, AR-PAM has achieved a lateral resolution of 45 μm and an imaging depth of 3 mm [41]. Dark-field PAM is able to monitor brain activity through the intrinsic blood hemodynamic response and can distinguish between increased blood oxygenation and a decreased blood volume *via* spectroscopic techniques. The ability of PAM to track blood oxygenation in the mouse brain was shown using a technique that controls hypoxic and hyperoxic changes [88]. Recently, the PAM technique has been applied to subcutaneous vasculature imaging [35], breast tumor detection [89], drug delivery [90,91], tissue temperature monitoring [92] and oxygenation monitoring in blood vessels [36,85].

Based on the advantages of PAM and excitation wavelength optimization, Liao *et al*. have successfully applied acoustic-resolution functional photoacoustic microscopy system (fPAM) to probe changes in CBV (Figure 5B) and SO_2_ (Figure 5C) without a labeling agent under electrically stimulation. This technique is ideal for *in vivo* imaging of changes in HbT, CBV and SO_2_ in single cerebral cortex vessels in small animals [37] (Figure 5D). The fPAM technique has a high resolution and can achieve a satisfactory imaging quality in small animals with good penetration depth. fPAM can be used visualize dynamic and functional activities in the brain because of the intrinsic optical absorption contrast of blood in brain tissues and the ability to distinguish between increased blood oxygenation and a decreased blood volume *via* spectroscopic techniques. Details of the method for selecting the proper wavelength for the intrinsic contrasting of optical absorptions have been described and discussed in a previous report [40]. This technique has been used to visualize 1) cerebral hemodynamic responses to neuronal activities induced by electrical stimulation in rats [40,86] (Figure 5E), 2) cerebral functions based on changes in HbT, CBV and SO_2_ in the rat brain [38] and 3) transcranial (through the cranium, through the skull) functional cerebral hemodynamic changes in single blood vessels [37,39]. In addition, Yao *et al*. proposed a method to measure transverse blood flow speed (Figure 5F) and velocity (Figure 5G) with directions that employs PA Doppler broadening of the signal bandwidth [87].

These authors confirmed that their method reliably measures the blood flow in the microvasculature of the mouse ear. These results suggest that all of the parameters which are needed for CMRO_2_ estimation (*i.e.*, the functional changes in cerebral HbT, CBV, SO_2_ and blood flow) are measurable *via* the current fPAM technique in one single setup. One future challenge is the exact quantification CMRO_2_*via* fPAM, as this will be very useful in clinical applications. Thus, transcranial monitoring of cerebral blood oxygenation by multi-wavelength PA system was reported recently. It was demonstrated that the oxygenation-related PA signal is detectable through the sheep’s intact scalp and skull, so the same technology can be performed in humans [93]. The skull strongly absorbs and scatters light, but the photon recycler increases the light transmittance through the bone. This promising technology had demonstrated the feasibility of photoacoustic tomography through the adult human skull [94].

Another future challenge is to design a “portable real-time fPAM system” for freely moving animals. Such a technique would be safe and would provide both a high contrast and high spatial resolution. Zemp’s group recently designed a portable PAM with an optical-level resolution and demonstrated the performance of this device in both *in vitro* and *in vivo* studies [95]. This handheld PAM retains the original merits of the previous OR-PAM system and allows real-time imaging to be performed. This fiber based handheld PAM design has the potential to be used in applications of freely moving animals.

### Functional near-infrared spectroscopy

The fNIRS technique relies on specific laser wavelengths (usually in the range of 700 – 1,700 nm) penetrating through the scalp to enable non-invasive measurement of changes in brain activity [43,44]. Although the spatial resolution of fNIRS is lower than that of other optical imaging techniques, fNIRS offers distinct advantages over fMRI, such as much lower cost, portability, ability to make continuous and long term measurements, and the ability to separate the relative concentrations of SO_2_, Hb and HbT when several proper wavelengths are simultaneously used. Previous studies have applied NIRS in functional imaging and cortical brain mapping based on the observation that regional CBF increases in response to a stimulus event. A variety of methods have been developed for fNIRS instruments, including the 1) continuous wave (CW), 2) time domain (TD) and 3) frequency domain (FD) methods, which have been applied in functional imaging to map motor [96], visual [8] and resting-state connectivity [43] in small animal models.

The CW method is used to measure changes in the incident light amplitude [44]. The laser light source is emitted and illuminates a selected scalp surface position. After the light has passed through the skull near the brain surface, highly scattered and attenuated light can be observed. Therefore, light intensity changes can be measured from a few centimeters below the skull. The intensity changes at the selected light wavelengths can be used to calculate the concentrations of HbO and HbR based on the Beer-Lambert Law (BLL) [44]. Because a CW system can only detect the amplitude decay of light, the exact volume of tissue that the light has illuminated is unknown; therefore, the absolute values of the hemodynamic concentrations cannot be provided [44]. In contrast, TD- and FD-fNIRS systems can estimate the path length of the NIR light from multi-distance sensors to derive the absolute values of hemodynamic concentrations [45]. For example, time-resolved near-infrared spectroscopy (TR-NIRS) systems employ short, picosecond, laser pulses and a fast photon-counting detector to detect the flight time of photons as light is attenuated through various cerebral tissue layers (*i.e.*, the skull) [97]. In addition, the TR-NIRS technique is comparable to IOS because of its’ ability to distinguish changes in absorption from changes in scattering [97].

A future challenge is to improve the fNIRS technique for use in clinical settings and studies, such as for rehabilitation or for brief evaluations before and after a trial of a particular therapy [98,99]. For example, although examining an individual stroke survivor’s unique brain response might be one method to determine the appropriate rehabilitation program, the use of fNIRS may provide a better understanding of the brain reorganization and motor recovery that have occurred and could provide a new avenue for designing therapeutic rehabilitation strategies that are tailored to the individual. We expect many unique and significant discoveries to be made in the area of brain-based rehabilitation research. Due to its many advantages, including cost, convenience of noninvasive and real-time imaging, etc., clinical studies applying fNIRS are likely to find use and applications in the very near future.

## Multimodal imaging techniques

The use of only one imaging technique to study cerebral functions provides a descriptive view of a single aspect of the underlying multi-faceted physiological processes. Multimodal imaging techniques provide a better way to study complex cerebral functions by allowing simultaneous measurement of multiple relevant physiological and biophysical parameters. Several multimodal fPAM techniques for neuroimaging have been proposed recently. For example, Wang *et al*. described a dual-modality microscope that combines PAM and fluorescence confocal microscopy (PA-FCM), designed for use in *in vivo* SO_2_ imaging [100]. Tan *et al*. proposed a multimodal imaging system that combines laser-scanning optical-resolution photoacoustic microscopy (LSOR-PAM) with spectral-domain optical coherence tomography (SD-OCT) to obtain better structural information along with that of blood vessels [101]. They demonstrated the feasibility of measuring *in vivo* metabolic changes in small animals. To achieve molecular-specific imaging with a high spatial resolution in deep tissue, Yakovlev*, et al*. combined molecularly specific stimulated Raman excitation with photoacoustic detection [102]. They found that unique stimulated Raman photoacoustic waves can be triggered and detected using a traditional ultrasonic transducer to form an image, which follows the design of the established PAM technique.

The combination of different imaging techniques in the same session seems very promising. For example, PA that is agent–free imaging method, was successfully indicates glucose metabolism level [103]. Fluorescently-labeled deoxyglucose analog, called 2-(N-(7-nitrobenz-2-oxa-1,3-diazol-4-yl)amino)-2-deoxyglucose (2-NBDG), which penetrates across the BBB, provided significant contrast for the photoacoustic imaging [103].

VSDi was successfully used for a neurovascular coupling studies in the epileptic research. Thus, simultaneous monitoring of the cerebral blood volume and VSDi demonstrated that an epileptic seizure consist of multiple dynamic multidirectional waves of membrane potential change that spread through a widespread network [104]. It seems extremely promising that VSDi recently has been used in combination with LSCI to directly visualize cortical spread depression. LSCI was employed for the monitoring of the CBF changes while VSD was used for direct recording of the neural activity [105].

Another powerful imaging method is the combination of VSDi with 2-photon excitation effect. This combination allows depth-resolved optical recordings with high temporal resolution not only from anesthetized but also from awake animals [24]. Theoretically, 2-photon excitation can be combined with many types of fluorescence probes, but it is possible to use such combination only in animal experiments due to the effects of fluorescence agents such as bleaching and cytotoxicity.

The fluorescent optical biomarker, indocyanine green (ICG), has been employed in combination with fNIRS [106]. This method not only brings new data about neurovascular coupling but also may be of wide clinical interest, since ICG is non-toxic as and generally considered safe (and approved for human use by the Food and Drug Administration). ICG is already used in clinical applications, such as retinal imaging, and hence in combination with various imaging modalities, it may prove to be valuable in performing imaging studies at the bedside in patients.

## Translation to human studies

Transcranial optical imaging remains challenging since optical scattering and absorption degrade the spatial resolution as well as the signal-to-noise ratio [103]. Brain imaging based on fluorescence probes was first conducted in 1948 [107] but was not considered an established method until recently. Fluorescence angiography provides a method to visualize perfusion during neurovascular operations, such as the treatment of angioma and the clipping of aneurysms. The fluorescent dye employed in this technique; usually ICG binds to plasma proteins and remains intravascular. It was successfully employed during neurosurgery [108] for the vascular imaging. However, this dye fails to reveal relative microcirculatory flows or tissue perfusion. Other dyes, such as aminolevulinic acid (ALA)-induced protoporphyrin IX (PpIX), bind selectively to tumor cells to facilitate the resection of brain tumors. Angiogenesis is essential for brain tumor development therefore the changes in the local neurovascular coupling play important role in the tumor localization and anticancer therapy. Brain glucose metabolism, one of the most important factors in the neurovascular coupling, can be studied using specific fluorescent glucose substitutes [109]. In clinical practice, 2-deoxy-2-[(7-nitro-2,1,3-benzoxadiazol-4-yl)amino]-D-glucose (2-NBDG) has been used frequently due to its strong fluorescence and low toxicity [103].

Advances in brain imaging can also contribute to the understanding of cognitive phenomena and neurological diseases [78], as these may correlate well with the changes in the neurovascular –coupling. Cerebral blood measurements are of great importance in the clinical setting especially in identification of progressive hypoperfusion before the onset of permanent brain damage [110]. But the imaging of the CBF as well as local blood and tissue oxygenation did not exist in the clinical practice twenty years ago. Regional changes in CBF, local oxygenation and metabolism have been reported in epilepsy, depression, bipolar disorder, brain trauma and other types of the brain pathology. These measurements are not only useful in a variety of surgical procedures, such as during clipping of aneurysms or vessel bypasses to assess baseline blood flow but also during neural or glial tumor resection to assess postsurgical tissue viability. Such measurements provide information allowing the identification of motor, sensory and speech activation centers in the cortex in procedures that require functional localization. However, the application of optical imaging techniques in humans presents several difficulties.

Due to the spatial resolution limitations imposed by the thickness of the human skull and the dura mater, beyond the first few years of life, the use of transcranial optical imaging can be performed only on the exposed brain during the course of therapeutic neurosurgical interventions. Because calcium- and voltage-sensitive dyes are not approved for use in humans, most studies have relied on intrinsic signals. The IOS is based on the light absorption and reflection property changes that occur in neural tissue when activated [111]. Although the spatial resolution (up to ~50 μm) of the IOS is pretty good for the human studies, IOS lucks the depth resolution in contrast with fMRI. Anyway, it is suitable for many functional studies that can utilize functional optical changes arising at the cortical surface. The temporal resolution of the IOS is better than that of fMRI in many cases. Furthermore, the affordable IOS setup is more affordable as it requires no additional modification of the operating room (OR). It may also be safe as no physical contact with tissue is required, thus avoiding potential risk of infection. Because light scattering and absorption are determined by the wavelength of the photon involved, the use of infrared and near-infrared light allows imaging at greater depths. For this reason, NIRS has been utilized extensively in clinical situations, including in non-invasive studies in infants with thin skulls [81]. Because the NIRS apparatus is portable and tolerable to movement by the subject during image acquisition, this technique has proven to be successful for many applications, such as the localization of epileptic seizures with increasing local cortical blood circulation [112] and in cognitive neuroscience research [113]. Based on the light-scattering pattern produced upon neural activation, which is likely due to optical changes caused by ionic movements across the neuronal membrane [10], a similar imaging technique referred to as the event-related optical signal (EROS) has been used in numerous functional studies [114]. Intraoperative imaging using EROS in awake patients during brain surgery has allowed characterization of stimulation-evoked epileptiform activity and the activation of Wernicke’s and Broca’s areas during language tasks [112,115]. Based on initial reports as well as its high temporal resolution and real-time capabilities, LSCI might be an efficient method to image CBF intraoperatively, with minimal interference to the ongoing surgery.

Although PA imaging presents many advantages [116,117] application of this technique is also faces several difficulties. For example, because physical contact is needed for efficient ultrasound transmission, use of a sterile sleeve to cover the transducer and sterile saline solution as a coupling medium are imperative. Nevertheless, because of its high resolution and ability to carry out multifunctional analysis of brain structure and metabolism, PAM appears to be a promising tool for measuring cerebral hemodynamic responses during neurovascular procedures.

It may take a while before a definitive technique for intraoperative neurovascular imaging is developed. Ideally, a suitable neurovascular imaging technique should have both high spatial and temporal resolution and should allow itself to be incorporated harmoniously within surgical techniques. The alternatives presently available allow compatible integration within the OR setting, precise anatomical resolution, or real-time accuracy, but not all three simultaneously. However, several technological advances suggest that an ideal system is possible in the future. Features such as fiber-optic and microscope miniaturization allow imaging techniques, previously exclusive to the bench top, to be made portable [118]. The use of a single optical fiber as both the illumination point source and detection pinhole, in combination with tissue-specific fluorophores and handheld confocal systems, is currently being tested in neuroimaging and brain tumor surgery [119]. A synergy between these high-resolution optical systems and the other systems reviewed above may provide definitive surgical imaging tools in the near future.

Electroencephalography (EEG) is often considered the "golden standard" in neurology. However, EEG monitoring is inadequate for questions regarding co-localization of electrical activity and changes in oxygenation and blood flow resulting from metabolic activity [120-122]. Thus, for such applications and measurements optical recordings may actually be more adequate and also applicable to monitoring or localizing foci of particular neurological disorders. The goal of the current brain optical imaging studies will be to translate recent findings into clinical practice.

## Conclusions

We have reviewed a wide range of *in vivo* optical imaging techniques and explored their many important applications in studying the cerebral neurovascular functions. We have further provided insight into the fundamental basis of many *in vivo* optical imaging techniques and highlighted important considerations for their practical implementation. We hope that this detailed review will aid researchers who are interested in either using or developing *in vivo* optical imaging techniques to understand the key aspects that should be considered when acquiring measurements or analyzing data. We have surveyed the huge body of literature that discusses studies in which *in vivo* optical imaging technologies and systems have been used successfully in ground-breaking and fundamentally important research and applications in cerebral neuroscience. The development of *in vivo* optical imaging techniques represents a rapidly expanding field that is continually evolving to embrace new technologies and clinical applications.

## Competing interests

The authors declare that they have no competing interests.

## Authors’ contributions

Study concept and design (LDL, VT and NT); drafting of the manuscript (LDL and VT); critical revision of the manuscript for important intellectual content (IDM, MLL, RE, AV, JO, NT and LDL); obtained funding (NT, LDL and YRL); administrative, technical, and material support (HYL, YYC and NT, LDL); study supervision (LDL and NT). All authors read and approved the final manuscript.
